# Nutritional rehabilitation in anorexia nervosa: review of the literature and implications for treatment

**DOI:** 10.1186/1471-244X-13-290

**Published:** 2013-11-07

**Authors:** Enrica Marzola, Jennifer A Nasser, Sami A Hashim, Pei-an Betty Shih, Walter H Kaye

**Affiliations:** 1 Department of Neuroscience, Section of Psychiatry, University of Turin, Turin, Italy; 2 Department of Nutrition Sciences, Drexel University, 19102 Philadelphia, PA, USA; 3 Department of Medicine, St. Luke's/Roosevelt Hospital Center, 10025 New York, NY, USA; 4 UCSD Department of Psychiatry, University of California, San Diego, 8950 Villa La Jolla Drive, Suite C – 207 La Jolla, 92037 San Diego, CA, USA

**Keywords:** Anorexia nervosa, Treatment resistance, Nutritional rehabilitation, Refeeding, Weight restoration, Weight maintenance, Caloric requirements, Refeeding syndrome

## Abstract

Restoration of weight and nutritional status are key elements in the treatment of anorexia nervosa (AN). This review aims to describe issues related to the caloric requirements needed to gain and maintain weight for short and long-term recovery for AN inpatients and outpatients.

We reviewed the literature in PubMed pertaining to nutritional restoration in AN between 1960–2012. Based on this search, several themes emerged: 1. AN eating behavior; 2. Weight restoration in AN; 3. Role of exercise and metabolism in resistance to weight gain; 3. Medical consequences of weight restoration; 4. Rate of weight gain; 5. Weight maintenance; and 6. Nutrient intake.

A fair amount is known about overall caloric requirements for weight restoration and maintenance for AN. For example, starting at 30–40 kilocalories per kilogram per day (kcal/kg/day) with increases up to 70–100 kcal/kg/day can achieve a weight gain of 1–1.5 kg/week for inpatients. However, little is known about the effects of nutritional deficits on weight gain, or how to meet nutrient requirements for restoration of nutritional status.

This review seeks to draw attention to the need for the development of a foundation of basic nutritional knowledge about AN so that future treatment can be evidenced-based.

## Introduction

Anorexia nervosa (AN) is a complex and frequently intractable illness of unknown etiology that is often chronic and disabling. It is characterized by aberrant feeding behaviors, an extreme pursuit of thinness and emaciation, and body image distortions. Onset tends to occur in females during adolescence and up to 0.7% of this age group may be affected, [1,2], while the current lifetime prevalence estimate of this illness is 0.3% [3]. Two subtypes of eating-related behaviors in AN are typically described. First, restricting-type anorexics (R-AN) lose weight purely by dieting and exercising without binge eating or purging. Second, binge-eating/purging-type anorexics (BP-AN) also restrict their food intake and exercise to lose weight, but periodically engage in binge eating and/or purging.

AN is often associated with denial of illness and resistance to treatment. Consequently it is difficult to engage individuals with AN in treatment, including nutritional restoration, and weight normalization [4]. The continuous restrictive eating and malnutrition result in pervasive disturbances of most organ systems including cardiovascular and gastrointestinal complications, endocrine disorders (i.e. osteopenia and amenorrhea), and other metabolic alterations [5]. Thus, nutrition restoration is a core element in treatment because of the need to restore weight [4] in order to avoid severe physical complications and to improve cognitive function to make psychological interventions useful and effective [6].

Individuals with AN often drop out from treatment programs and relapse because of continued harmful eating behaviors [7]. They tend to have frequent hospitalizations and chronicity and high mortality [8,9]. Aside from resistance and denial, there are other contributory factors. First, data suggest that individuals with AN, particularly restricting type, have difficulty eating because it generates a profound sense of anxiety, instead of pleasure or reward [10]. There is emerging evidence that patients with AN have alterations in neural mechanisms that would normally activate the drive for food consumption when starved or hungry [11]. In addition, patients with AN tend to consume vegetarian diets more often than the general population [12]. Given the weight-related nature of their motivation for vegetarianism [12], their diet results in low calorie and low fat meals insufficient for daily calorie [13], essential fatty acid, and amino acid [14] requirements. Even in weight-restored patients abnormal eating behavior often persists, as limited diet variety was found to be associated with poor outcomes [15]. From a clinical standpoint, AN individuals appear to become hypermetabolic during weight restoration, [16,17] such that they need an increased caloric intake to maintain a safe weight after recovery [16,18]. The tendency to return to restrictive eating habits after hospitalizations compounds the need for increased caloric requirements due to hypermetabolic state [19].

There is limited empirical data available that define optimum food choices for weight restoration and address the challenges associated with rate of weight gain and weight maintenance. This has hampered the development of evidence-based guidelines for nutritional rehabilitation therapy in AN. This review aims to summarize what is known about caloric and nutritional requirements to gain and maintain weight in patients with AN. It also highlights the urgent need to expand basic nutritional knowledge pertaining to AN in order establish evidenced based treatments.

### Methods

For the purpose of this review we conducted a PubMed search (date 1960 to 2012) to identify and evaluate published English language papers on caloric requirements for weight restoration in AN. Inclusion criteria were: a) English language, and b) date ranging from 1960 to 2012. The search string 'anorexia nervosa AND caloric requirements OR caloric intake OR weight restoration OR refeeding OR nutritional rehabilitation’ was applied. This yielded 1,144 titles, of which a more detailed abstract and full-text search was conducted to exclude not strictly related papers. In addition, both APA and NICE guidelines were considered.

## Review

### AN eating behavior

It is well known that patients with AN, compared to healthy controls, tend to eat significantly fewer calories [13,20-22] by restricting caloric intake [23] and avoiding calorie dense foods [24]. Individuals with AN show many unusual eating behaviors like slow and irregular eating [25,26], vegetarianism [12,23,26,27], and choosing a narrow range of foods [23,28]. Interestingly, it has been demonstrated that these disordered eating patterns are present before the onset of illness. Affenito et al. [21] described how daily diets of premorbid individuals (one year before the full diagnosis of AN) are significantly less calorie dense than those of healthy controls. Consuming only low energy-dense vegetarian food as a means of losing weight [12] may create a number of problems [29] such as a severe deficit in essential nutrient intake when plant based sources of proteins are not properly balanced resulting in the lack of one or more essential amino acids and inadequate protein synthesis in the body. It is interesting to note that voluntary caloric restriction in lean individuals, involving ingestion of only 75% of daily caloric requirements, for the purpose of improving longevity and slowing aging, is not associated with a severe deficit in essential nutrient intake, most likely due to the inclusion of adequate amounts of diverse protein and micronutrients in the diet prescription [30].

In studies evaluating the differences in food consumption between AN patients and healthy controls, adolescents with AN showed a lower intake of all types of fat, higher fiber intake, and normal proteins and carbohydrates in one study [20], while a lower intake of fat but higher intake of carbohydrate and no difference in protein was found in another study using a naturalistic laboratory study design [22]. Studies in adult AN patients are in line with adolescent data showing macronutrients and micronutrients deficiencies (see Tables 1 and 2); several studies demonstrated how fatty foods are widely avoided [20,22,23] and how total energy intake is lower in AN than in healthy controls [13,31]. In terms of carbohydrates, some studies have found an increased percentage intake in AN diets [22,23] but other studies did not confirm these data [13,21,31-33]. Similarly, data on percentage of protein intake in AN patients are inconsistent; it was found to be increased in some studies [22,23] but decreased in others [13,32,33].

**Table 1:** Overview of studies conducted on % macronutrients intake in adolescents and adults with anorexia nervosa

	**Sample**	**% Fats**	**% Carbohydrates**	**% Fibers**	**% Proteins**
**Misra, 2006 **[20]	Adolescents	↓		↑	
**Hadigan, 2000 **[23]	Adults	↓	↑		↓
**Fernstrom, 1994 **[22]	Adults	↓	↑		=
**Beaumont, 1981 **[31]	Adults	↓	=		↑
**Jáuregui Lobera, 2009 **[33]	Adults	↓	↓	=	↓
**Gwirtsman, 1989 **[13]	Adults	↓	↓		↓
**Affenito, 2002 **[21]	Adolescents	↓			
**Russell, 1967 **[32]	Both	↑	↓		↑

**Table 2:** Overview of studies conducted on micronutrients intake in adolescents and adults with anorexia nervosa

	**Misra 2006 **[20]	**Hadigan 2000 **[23]	**Beaumont 1981 **[31]	**Jáuregui Lobera 2009 **[33]
**Sample**	**Adolescents**	**Adults**	**Adults**	**Adults**
Vit A	↓			
Vit K	↓			
Vit D	↓	↓		
Vit B12	↓	↓		↓
Vit C			↓	
Vit B6	↓			
Calcium	↓	↓	↓	
Zinc	↓	↓		↓
Folate	↓	↓		
Niacin			↓	
Sodium				↓
Phosphorus				↓
Copper				↓
Selenium	↓			↓
Megnesium	↓			
Iron	↓			
Thiamin	↓			
Riboflavin	↓			
Pantothenic Ac	↓			
Retinol			↓	

It has also been reported that AN patients tend to choose the same types of foods at each meal [23] and these eating behaviors persist during short-term recovery; in fact low energy dense food and limited variety were associated with poor outcome [15]. It has been recently shown that high dietary energy density scores are more predictive of better outcomes than total caloric intake [15,34]. A follow-up study of food intake one year after hospital discharge showed that individuals with AN tend to revert to pathological eating and to the low calorie intake [19].

In terms of actual caloric intake, it should be noted that healthy young adult women tend to eat about 30 kilocalories/kilogram per day (kcal/kg/day), with a range of 20 to 40 kcal/kg/day [35]. For a 50 kg women this means eating 1,500 kcal/day with a range of normal of between 1,000 and 2,000 kcal/day. In our experience, individuals with AN tend to find it difficult to eat more than 10 to 20 kcal/kg per day (30 kg = 300 to 600 kcal/day).

### What is known about weight restoration in AN?

Nutritional and weight restoration is a core component of many treatment programs for AN. Despite this emphasis, there has been relatively little research in this area in AN. This is all the more notable when considering that there is a substantial literature on refeeding after forced starvation or a prolonged fast in non-AN individuals [36-38].

Both APA [4] and NICE [39] guidelines specify clearly how the first goal of treatment is weight restoration [4,39] (Tables 3 and 4). But APA guidelines do not specify caloric intake guidelines for outpatients; in fact they suggest amounts related to hospitalization, and there is no mention of the quantity and quality of nutrients that are most critical to achieve treatment goals. NICE guidelines report the weekly weight gain that can be expected both in AN inpatients and outpatients, but specific caloric prescriptions are not included. The literature on the importance of gaining weight in AN [40-42] includes refeeding in severe and resistant AN cases [43,44], clinical improvement requiring caloric intake [25,45], effects of micronutrients deficiencies and alterations [46] on adolescent patients [47-51], inpatient treatments [52] and risks during refeeding [53,54]. However, relatively little is known about diminished essential nutrients or what food products are most useful in replenishing such essential nutrients. This issue is confounded by the fact that dietary choice in AN is driven by preference of vegetarian-based, low energy-dense diet of food type, rather than a complete starvation mode.

**Table 3:** American Psychiatric Association (APA) guidelines for anorexia nervosa

***Nutritional Rehabilitation***	
The goals of nutritional rehabilitation for seriously underweight patients are to restore weight, normalize eating patterns, achieve normal perceptions of hunger and satiety, and correct biological and psychological sequelae of malnutrition.	I
In working to achieve target weights, the treatment plan should also establish expected rates of controlled weight gain. Clinical consensus suggests that realistic targets are 2–3 pounds (lb)/week for hospitalized patients and 0.5-1 lb/week for individuals in outpatient programs.	II
Registered dietitians can help patients choose their own meals and can provide a structured meal plan that ensures nutritional adequacy and that none of the major food groups are avoided.	I
It is important to encourage patients with anorexia nervosa to expand their food choices to minimize the severely restricted range of foods initially acceptable to them.	II
Caloric intake levels should usually start at 30–40 kilocalories/kilogram (kcal/kg) per day (approximately 1,000-1,600 kcal/day). During the weight gain phase, intake may have to be advanced progressively to as high as 70–100 kcal/kg per day for some patients; many male patients require a very large number of calories to gain weight.	II
Patients who require much lower caloric intakes or are suspected of artificially increasing their weight by fluid loading should be weighed in the morning after they have voided and are wearing only a gown; their fluid intake should also be carefully monitored.	I
Urine specimens obtained at the time of a patient's weigh-in may need to be assessed for specific gravity to help ascertain the extent to which the measured weight reflects excessive water intake.	I
Regular monitoring of serum potassium levels is recommended in patients who are persistent vomiters.	I
Weight gain results in improvements in most of the physiological and psychological complications of semistarvation.	I
It is important to warn patients about the following aspects of early recovery:	I
As they start to recover and feel their bodies getting larger, especially as they approach frightening, magical numbers on the scale that represent phobic weights, they may experience a resurgence of anxious and depressive symptoms, irritability, and sometimes suicidal thoughts. These mood symptoms, non-food-related obsessional thoughts, and compulsive behaviors, although often not eradicated, usually decrease with sustained weight gain and weight maintenance. Initial refeeding may be associated with mild transient fluid retention, but patients who abruptly stop taking laxatives or diuretics may experience marked rebound fluid retention for several weeks. As weight gain progresses, many patients also develop acne and breast tenderness and become unhappy and demoralized about resulting changes in body shape. Patients may experience abdominal pain and bloating with meals from the delayed gastric emptying that accompanies malnutrition. These symptoms may respond to pro-motility agents.	III
When life-preserving nutrition must be provided to a patient who refuses to eat, nasogastric feeding is preferable to intravenous feeding.	I

Legend:

I: Recommended with substantial clinical confidence; II: Recommended with moderate clinical confidence; III: May be recommended on the basis of individual circumstances.

**Table 4:** National Institute for Clinical Excellence (NICE) guidelines for anorexia nervosa

***Managing weight gain in AN***	
In most patients with anorexia nervosa an average weekly weight gain of 0.5 to 1 kg in inpatient settings and 0.5 kg in outpatient settings should be an aim of treatment. This requires about 3,500 to 7,000 extra calories a week.	C
Regular physical monitoring, and in some cases treatment with a multivitamin/multi-mineral supplement in oral form is recommended for people with anorexia nervosa during both inpatient and outpatient weight restoration.	C
Total parenteral nutrition should not be used for people with anorexia nervosa, unless there is significant gastrointestinal dysfunction.	C
***Managing risk in AN***	
Health care professionals should monitor physical risk in patients with anorexia nervosa. If this leads to the identification of increased physical risk, the frequency and the monitoring and nature of the investigations should be adjusted accordingly.	C
People with anorexia nervosa and their carers should be informed if the risk to their physical health is high.	C
The involvement of a physician or paediatrician with expertise in the treatment of physically at-risk patients with anorexia nervosa should be considered for all individuals who are physically at risk.	C
Pregnant women with either current or remitted anorexia nervosa may need more intensive prenatal care to ensure adequate prenatal nutrition and fetal development.	C
Oestrogen administration should not be used to treat bone density problems in children and adolescents as this may lead to premature fusion of the epiphyses.	C

Legend:

Evidence C: This grading indicates that directly applicable clinical studies of good quality are absent or not readily available.

**Figure 1:**
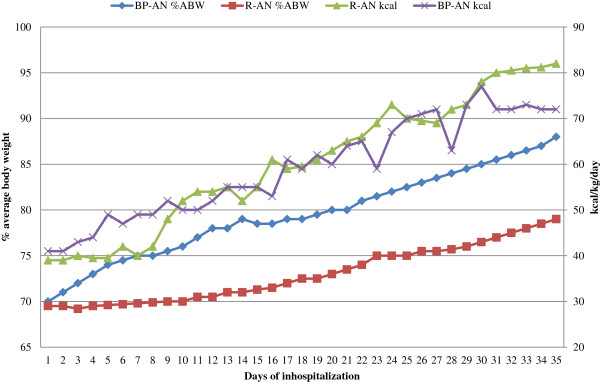
**Restricting-type anorexia nervosa (R-AN) patients need more kilocalories (kcal) than binge-purging-type AN (BP-AN) patients to gain the same amount of weight.** While individuals with restricting-type and binge-purging-type AN consume similar kcal per kilogram (kg) per day, those with restricting- type AN gain weight more slowly in terms of % average body weight (% ABW) (Kaye et al., unpublished data).

A number of studies have measured caloric intake during weight gain in AN and have estimated the amount of caloric intake needed to gain a kg of weight (Table 5) [8,25,44,45,55-59]. It should be noted that differences in caloric requirements have been reported between AN subtype groups. Kaye and colleagues showed that R-AN patients need more calories than BP-AN patients to gain an equal amount of weight [16] (Figure 1).

**Table 5:** Excess calories to gain weight in anorexia nervosa (kcal/kg of weight gain)

	**R-AN**	**BP-AN**	**AN**
**Walker et al., 1979 **[17]	6401 ± 1627		
**Newman et al., 1987 **[56]	4937.8 ± 1675	5324.1 ± 2457.3	
**Forbes et al., 1982 **[57]			4730 ± 540
**Russell and Mezey, 1962 **[58]			7525 ± 585
**Dempsey et al., 1984 **[55]			9768 ± 4212
**Kaye et al., 1988 **[45]			8301 ± 2272
**Sunday and Halmi, 2003 **[25]	3055	2788	
**Gentile, 2012 **[44]			3500–7000
**Mehler et al., 2010 **[59]			1800-4500

Legend:

R-AN: anorexia nervosa restricting subtype.

BP-AN: anorexia nervosa binge-purging subtype.

**Figure 2:**
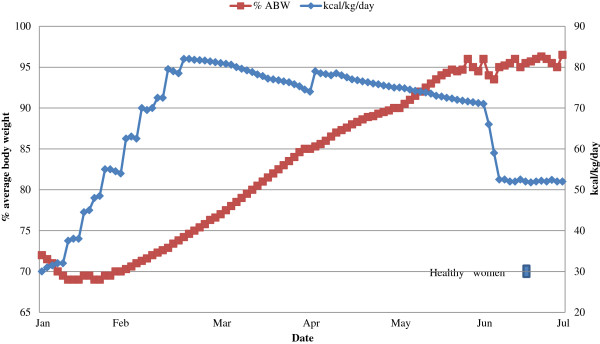
**Percent average body weight (% ABW) and kilocalories/kilogram per day (kcal/kg/day) in a typical course for a restricting-type anorexia nervosa individual who entered at 70% ABW.** Individuals with anorexia nervosa tend to require escalating caloric intake in order to maintain a 1 to 1.5 kg/week weight gain during hospitalization (Kaye et al., unpublished data).

In our experience, AN tends to require escalating caloric intake in order to maintain a 1 to 1.5 kg/week weight gain during hospitalization. Figure 2 illustrates a typical course for a restricting-type AN individual who entered at 70% average body weight (ABW). As noted above, healthy women without an eating disorder require approximately 30 kcal/kg/day to maintain their weight (range 20 to 40 kcal/kg/day). If refeeding for an individual with AN was started at this amount, they would ultimately fail to gain weight. Rather, their caloric intake would need to be increased, in steps over time, to somewhere between 60 to100 kcal/kg/day to show sustained weight gain.

### Role of exercise and energy metabolism in resistance to weight gain

It should be noted that there is evidence that people who are obese and lose weight become hypometabolic. That is, they may reduce their caloric intake but they have trouble losing weight or maintaining lost weight after a while [60,61]. Moreover, if they increase their food intake, they gain weight easily. People with AN seem to have an opposite problem. That is, they become hypermetabolic. They easily lose weight, and need to eat an even larger amount of food to gain weight [16,62]. As described below, caloric intake has a reduced efficiency in terms of being converted into tissue in AN patients [63]. Moreover, it has been shown that patients with AN who were previously obese can gain weight more rapidly than those patients without any history of obesity. This suggests that differences in metabolic rates may play a key role in the outcome of weight-gain effort [17].

**Figure 3:**
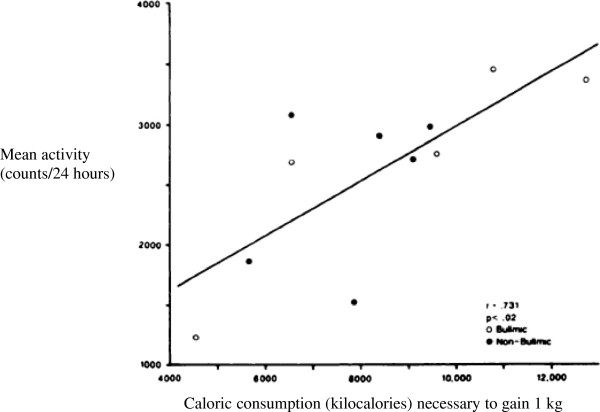
**Relationship between physical activity counts per day and the amount of kilocalories needed to gain each kilogram (kg) of body weight.** Exercise contributes to approximately a threefold range of calories to gain 1 kg of body weight (Kaye et al., 1988 [45], republished with permission).

Excessive exercise is a common behavior exhibited by many with AN [64]. In spite of severe emaciation, many continuously stand, or have restless motions, or even spend a considerable portion of the day pacing or jogging. Such exercise could contribute to the increased caloric requirements for weight gain [16]. Literature on the caloric expenditure during exercise in emaciated patients is scarce. Kaye and colleagues (Figure 3) showed the amount of exercise in AN individual engages in resulted in an almost threefold range of calories required to gain 1 kg [45]. That is, people who did little exercise only needed an excess of 4,000 calories to gain a 1 kg of weight, where as those who engaged in extreme exercise needed up to 12,000 additional calories to gain the same weight.

**Figure 4:**
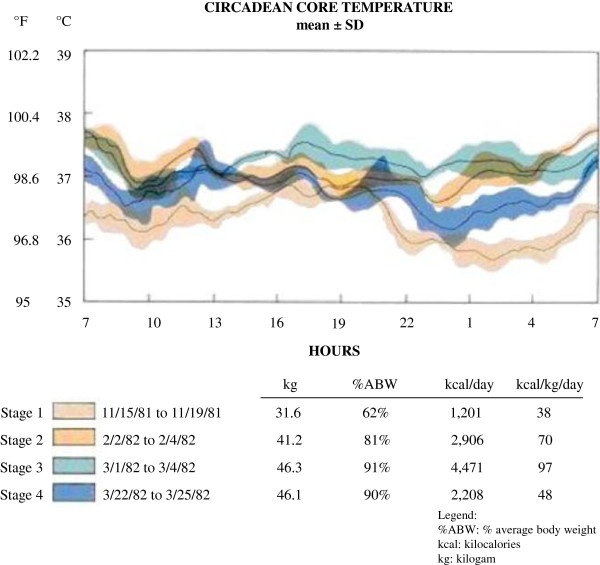
**Mean 24-hour core body temperature in individuals with anorexia nervosa (AN) at stages of weight restoration.** Increased caloric consumption is associated with an increase in core body temperature. At peak caloric intake (i.e. stage 3) AN patients lose the normal night time drop in body temperature; their temperature at night is elevated and they report being diaphoretic at night when asleep (Kaye et al., unpublished data).

There is also evidence that energy intake may be converted into heat, rather than being used to build tissue in AN. Our clinical experience (Figure 4) is that AN patients often complain of becoming hot and sweaty during nutritional restoration, particularly during the night. It is not uncommon that they will wake up sweating and their sheets are soaked. In fact, in a study of 24-hour circadian body temperature, we found evidence that AN individuals became hyperthermic (have elevated body temperature) that was most marked during the night, a time when body temperature decreases. This was not due to exercise as a body activity monitor confirmed they were sleeping. This notion is supported by studies showing that the thermic effect of food in AN patients during renutrition is high, [63,65,66] representing up to the 30% of energy expenditure instead of the 14-16% in healthy controls [67] and being particularly high at the beginning of refeeding [65]. Stordy et al. [68] have shown that metabolic rate of AN patients who were previously obese before the onset of AN was lower than the ones with no history of obesity during refeeding. The same study found that the patients who had experienced obesity also experienced a smaller thermic effect of refeeding than AN patients with no history of obesity, though still higher than healthy controls. The increased diet-induced thermogenesis can be explained both by the higher energy intake during refeeding and the low efficiency in the initial phases of nutritional restoration [63]. It is possible that the enhanced thermic effects of food during weight gain could be related to changes in hormones or autonomic function [67,69-72].

An important aspect of metabolism to consider during refeeding is the 6-fold greater energy requirement needed for gaining fat mass versus fat-free mass [73]. It is possible that during nutritional restoration more fat-free mass is initially synthesized in those with Body Mass Index (BMI, expressed in kg/m^2^) between 13 and 14 compared to those patients with BMI > 14 [73]. The importance of restoring fat mass is highlighted by studies showing that lower percentage of body fat, i.e. ≤ 4 kg, that corresponds with a BMI of 13, are related to higher resistance to treatments [74], poor prognosis [75], and death [76].

### Medical consequences of weight restoration

It is well known that emaciation is associated with substantial medical complications as previously described [5,77,78]. For example, many with AN have compromised cardiovascular status and shifts in fluid balance, with some being dehydrated and some overhydrated; reduced blood levels of albumin and anemia. If parenteral (intravenous) or enteral (tube) refeeding is necessary because of extreme resistance, administration of nutrients should be done slowly, starting with no more than 500 kcal/day in the form of a complete liquid diet for several days, then gradually increasing the caloric load in a step-wise matter. According to NICE guidelines [39] people at high risk are those with BMI < 12, those who vomit, abuse laxatives and binge and those with physical comorbidity [39]. In such severe cases, strict monitoring is required, and it may take a month or more to restore body weight, not necessarily to normal weight, but to an acceptable level (usually a 10% gain in weight) that can be followed by oral feeding on an outpatient basis.

One important side effect to be considered at the beginning of nutritional therapy - even if it is rare - is refeeding syndrome caused by rapid refeeding of someone in a state of starvation, usually chronic. It is well known that starvation to the level of 50% reduction in the normal body weight is associated with higher fatality rate [79]. Under these conditions, restoration of nutrition must be done slowly in order to avoid refeeding syndrome [80,81]. The adverse effects of rapid acute refeeding have been known since the experience with rehabilitation of concentration camp survivors [82]. This syndrome is biochemically characterized by hypophosphatemia, hypomagnesemia, hypokalemia, glucose intolerance, fluid overload, and thiamine deficiency. Clinical consequences can be cardiac arrhythmias, congestive heart failure, hypotension, respiratory failure, rhabdomyolysis, coma, seizures, skeletal-muscle weakness, encephalopathy, metabolic acidosis, and ataxia [80,81]. To avoid refeeding syndrome, levels of phosphorus, magnesium, potassium and calcium should be determined for the first 5 days and every other day for several weeks; electrocardiogram (EKG) should be also performed [4]. If indicated, during the first days of refeeding, large amounts of multi-vitamins and minerals, in particular potassium, thiamine, phosphate and magnesium, should be provided [39]. Again, strict monitoring is needed to prevent vitamin A and D toxicity in case of excessive supplements [39].

Long periods of malnutrition cause physical changes in the muscles, the integrity of the gut wall and digestive enzyme systems of the gut, as well as compensatory metabolic changes in the body to deal with being malnourished [83-85]. As a result, increases in caloric intake can cause gut distress because the body requires time to adapt to the processing of the increased food intake. Rapid increase in calories may result in dangerous conditions in some patients, therefore careful medical monitoring during the weight restoration process is required.

### Rate of weight gain

As noted earlier, there is very limited research regarding the rate of weight gain during refeeding. Thus, guidelines tend to be based on clinical experience, rather than evidence from research studies. Still, there is considerable data indicating that AN patients need somewhere between 5,000 and 10,000 excess calories to gain a kg of weight (Table 5). The reason why this range is so wide remains unclear. Nevertheless, several factors have been called into question to try to explain this variability: physical activity, individual variations in energy efficiency, thermoregulatory response, composition of synthetized tissue, fluid shifts, age, and phase of treatment [17,45,55,56].

We can estimate that, on the average, 7,500 kcal is required to gain a kg of weight (or 3,500 kcal to gain 0.5 kg of weight). In other words, to gain 1 kg (2 pounds, lb) a week, this means approximately an additional 1,000 kcal per day is needed. If an AN individual who is 35 kg (77 lb) needs about a 1,000 kcal per day (30 kcal/kg) to maintain her weight (it may be more – see below), than they would need to eat a minimum of 2,000 kcal/day to gain weight. However, in our clinical experience, this is a conservative estimate, most AN patients need to eat much more to achieve the weight goal because of both metabolic changes and partial compliance to treatment plans (i.e. skipping meals, purging behaviors).

With this perspective, we recommend at the start of weight gain, the caloric intake should be of 30–40 kcal/kg/day for inpatients because the first goal is safe clinical stabilization before beginning weight gain [4,62]. For outpatients the initial caloric intake should be approximately 20 kcal/kg/day. It is also very important to emphasize that because caloric intake (both the anticipation and real-time experience) generates heightened anxiety in those with AN, assurance of even these modest levels is problematic. The issues related to balancing the cost of treatment, anxiety and resistance, and the need for aggressive restoration are complex and are beyond the scope of this review [86-88].

Later in the refeeding process it is possible to gain about 1–1.5 kg/week for inpatients and 0.5 kg/week in outpatient therapy [4]. Studies suggest AN patients need an excess of about 3,400 kcal to gain 0.5 kg per week. This is more than the amount of calories needed just to maintain weight. Over the course of 7 days, the amount required for weight gain is approximately an extra 500 kcal/day to that needed for maintenance. For example, in the case of a 30 kg woman, if maintenance is 30 kcal/kg/day = 900 kcal/day; to gain weight 500 kcal/kg/day should be added so the starting total caloric intake will have to be 1,400 kcal/day.

Unfortunately, AN patients most likely will not continue to gain weight only by adhering to the recommended formula: 30 kcal/kg/day maintenance + 500 kcal/day for weight gain. Rather, we have found that the maintenance amount of calories needs to be increased at intervals to continue weight gain. That is, to continue gaining 0.5 kg per week it may be necessary – according to our experience - to do a step-wise increase of 10 kcal/kg/day every 5 to 7 days if there are plateaus in gaining weight. Some individuals with AN may require even more energy to achieve weight restoration and thus need up to 70 to 100 kcal/kg/day [62]. So this may mean consuming 4,000 to 5,000 or more calories per day.

During the whole complex process of refeeding, it is important to observe the trend of weight changes over time (weekly) by documenting the weekly weight change, rather than just react to daily changes because weight can fluctuate daily due to fluid shifts and bowel movements.

### Weight maintenance

**Figure 5:**
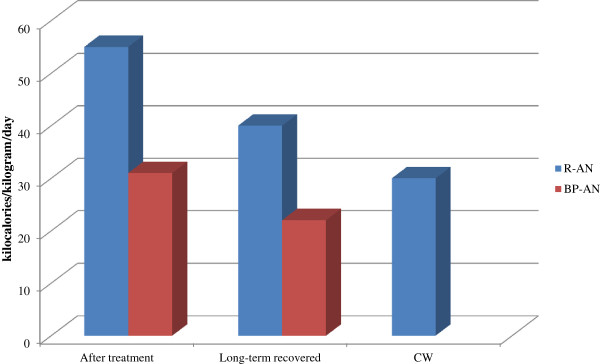
**Comparison of daily caloric requirements.** In the month after restoration of a healthy body weight, both restricting anorexics (R-AN) and binge-purging anorexics (BP-AN) remain energy inefficient when compared to individuals studied after long-term (>1 year) weight restoration or healthy control women (CW) ([13,16,18] original figure, no permission required).

Immediately after getting back to a healthy body weight, both R-AN and BP-AN are still highly energy inefficient and require increased caloric intake to maintain the restored weight (R-AN even more that BP-AN [16,18]) (Figure 5). In fact, if healthy women need 30 (20 to 40) kcal/kg/day for weight maintenance, the amount for weight maintenance is at least 50 to 60 kcal/kg/day for AN. The need of increased caloric requirement may be, in part, related to slow normalization of neuroendocrine processes [62]. Without this substantial amount of food, there is often rapid weight loss, which may partly explain the high rate of relapse, reported to be up to 50% in AN [9,16]. Eating attitudes upon hospital discharge represent a reliable predictor of outcome [89]. It has been reported that AN patients tend to regress back to an underweight body at the 1-year follow-up after hospitalization [19]. Psychological and physiological, as well as metabolic and neuroendocrine factors contribute to this serious obstacle to long-term recovery. It has been noted that the increased caloric needs cannot be explained by malabsorption [16,58]. Data in the scientific literature show that caloric needs tend to normalize with time [18]. It has been reported that over the course of 3 to 6 months, both R-AN and BP-AN show a normalization of their metabolism (needing between 20 and 40 kcal/kg/day to maintain weight), which is similar to the caloric amount needed by healthy women with no eating disorder[16,18,25]. To obtain the best chance of long-term weight maintenance recovery, AN patients should persist with an increased caloric intake treatment plan.

### Nutrient intake

The other issue, aside from the amount of food needed for nutritional restoration is the question as to what types of foods are best and/or acceptable to those with AN. Surprisingly, there has been very little research on this issue, despite the fact that nutritional rehabilitation is a core focus of most AN programs. From a nutrition standpoint, the chances of replenishing macro and micronutrient needs are enhanced by increasing the variety of foods that are prescribed for the patient with AN [15]. In reality, this represents a great difficulty for patients as resistance to eating a variety of foods is a core element of their symptomatology [23]. An important clinical issue is that no specific recommendations for macronutrient distribution in people with ED have been developed [62]. It should be noted that the daily macronutrient required (in adolescents and adults) to maintain weight (not gain), as defined by the Institute of Medicine, are 110–140 grams of carbohydrates, 15–20 grams of essential fatty acids and 1 gram of proteins per kilogram of body weight [90]. Recommended Daily Allowances (RDA’s) for vitamins and minerals vary by age and gender, but can be met by intake of a multivitamin/multimineral tablet or liquid. Placing the emphasis on nutrient requirements, achieved through food intake, as opposed to caloric intake, may help to lessen the anxiety and resistance to refeeding observed in AN patients.

It has been suggested that the AN patient should be eating calorie dense food to replenish the necessary nutrients [62,91]. Daily intake of foods containing protein of high biological value, such as whey and casein, and egg whites, that contain a high concentration of essential amino acids per gram and calorie density, should be recommended. Consuming small amounts of protein of high biological value, in conjunction with the protein source foods that are perceived as less challenging by AN patients (usually of a vegetable source), can help assure a faster restoration of nutrient status even in a continued state of reduced body weight. Additionally, a variety of protein food sources including fleshy fish and poultry should be encouraged.

Fat intake is a critical issue during AN refeeding; it is well known in scientific literature and in every-day clinical practice that AN individuals tend to avoid consuming fats [13,20,21] resulting in lipid depletion [14,92] and alterations [93]. Importantly, neuronal walls and wiring between brain regions is highly dependent on lipid function. It is particularly important that AN patients replenish these stores by eating lipids-rich foods during treatment. Many fatty acids can be produced endogenously, but linolenic acid, an omega-3 polyunsaturated fatty acid, and linoleic acid, an omega-6 polyunsaturated fatty acid, are essential fatty acids that must be provided in the diet. These essential fatty acids are crucial for cellular membrane function and gene regulation [94]. Eicosapentaenoic acid (EPA) is the precursor for eicosanoids and the docosahexaenoic acid (DHA) is a fundamental structural component of grey matter and retina. EPA and DHA are two important long-chain omega-3 fatty acids that can be found in high amount in fish, meat, and eggs. These are food sources that AN patients tend to avoid, making the need to replenish EPA and DHA even more critical. It has been demonstrated that levels of plasma long-chain omega-3 fatty acids in vegetarian and vegan individuals are lower than in meat-eaters [94]. In AN, enhancement of the biosynthesis of alternative fatty acids was found only partially compensated for the loss of polyunsaturated fatty acids [14]. It is interesting to note that use of an EPA derivative (ethyl-eicosapentaenoate, Ethyl-EPA) administered as a daily supplement at 1 gram/day for a 3 month period, in a small sample of R-AN patients, resulted in positive outcomes in terms of weight restoration [95].

A variety of carbohydrates can be offered, such as complex carbohydrates (bread, rice and potatoes) as well as fruits, fruit juices and vegetables. The choices made by the patient can be incorporated into the rest of the food ingredients. Vitamin and mineral RDA’s can be achieved through use of supplements in liquid or tablet form, that can augment the vitamin and mineral content found in foods.

The use of medical foods that are acceptable to the AN patient may also be considered when patients cannot eat a sufficient amount of food to achieve weight restoration or as a useful addition in case of unstable weight maintenance. Medical foods may reduce the stomach and gastrointestinal discomfort that refeeding with more caloric dense food may exaggerate. Moreover it has been showed that in AN patients, there is a delayed gastric emptying of solid but not of liquid meals [96,97], therefore liquid supplementations can be a well-tolerated intervention mostly at the beginning of refeeding treatment. To our knowledge, there is little research in this area, so the potential benefits remain to be proven, and the best food products remain uncertain.

## Conclusions

It is our clinical experience that the use of reason, insight, and intuition are of limited efficacy in convincing an individual suffering from AN to eat. If this is not true, there is little in the way of rigorously evidence in the literature to support such contentions with current practices of refeeding in AN being highly subjective and having limited backing in scientific research. In fact, our observations indicate that recommendations made by ED programs vary highly, and are dependent on the providers’ experience, resources, and biases, rather than research and evidence. This review serves not only as a synthesis of the current, though limited, research findings, but also to call for an urgent effort to improve treatment by stimulating such research.

In summary, we recommend that the restoration of both nutrient status and weight starts slowly and gradually accelerate as tolerated. There should be a continued focus on nutrient intake, as opposed to caloric intake, coupled with psychotherapy to encourage increasing both the amount and diversity in food selections with the eventual goal of weight and nutrition restoration in mind. Data of Schebendach and Colleagues [15] suggest that diet diversity is predictive of weight maintenance in AN patients. The emphasis on nutrient intake and status should provide a less anxiogenic approach to achieving increased dietary diversity, which should ultimately lead to consistent food intake levels capable of sustaining weight in the normal range.

Although nutritional restoration is a key-element in the treatment of anorexia nervosa, increased amounts of food also increase anxiety and resistance. Caloric requirements in AN patients are high and vary between 30–40 kcal/kg/day (up to 70–100 kcal/kg/day) for inpatients, and 20 kcal/kg/day for outpatients; after the first phase of treatment it is possible to achieve a weight gain of 1–1.5 kg/week in the inpatient setting and of 0.5 kg/week in the outpatient setting. Also, for maintenance, AN patients need higher caloric amounts - around 50–60 kcal/kg/day - than the general population. This increased caloric requirement may be due both to exercise – often a hallmark of this illness - and metabolism. In fact during the first phases of renutrition AN patients are very energy inefficient because they usually become hypermetabolic and show increased diet-induced thermogenesis as well as a variety of neuroendocrine alterations.

Treatment efforts during refeeding should focus on modifying the disordered dietary patterns that AN patients commonly practice, including slow and irregular eating, vegetarianism, and a restricted range of foods.

When severely malnourished, AN patients often need to be admitted to a hospital in order to receive more aggressive treatment, extra care and the monitoring required prevent the occurrence of refeeding syndrome. Regular monitoring of vitals, electrolytes and cardiac functions are critical. Caloric requirements and nutritional deficits continue to be critical issues in anorexia nervosa treatment and management. Much more research is needed in this area to better understand and optimize caloric intakes and refeeding practices for individuals recovering from AN, and to develop complete and reliable guidelines for clinicians and providers about this important topic.

## Abbreviations

AN: Anorexia nervosa; R-AN: Restricting anorexia nervosa; BP-AN: Binge-purging anorexia nervosa; ED: Eating disorder; APA: American Psychiatric Association; NICE: National Institute for Health and Care Excellence; m: Meter; kg: Kilogram; kcal: Kilocalories; ABW: Average body weight; BMI: Body mass index; RDA: Recommended daily allowances; EKG: Electrocardiogram; EPA: Eicosapentaenoic acid; DHA: Docosahexaenoic acid; CW: Control women.

## Competing interests

The authors have no competing interests to declare.

## Authors’ contributions

EM: was involved in the study design, made substantial contributions in the review process and drafted the manuscript; JAN: made substantial contributions in the review process and drafted the manuscript; SAH: made substantial contributions in the review process; PBS: critically revised the paper; WHK: conceived the study, made substantial contributions in the review process, drafted the manuscript and gave final approval to the version to be published. All authors read and approved the final manuscript.

## Pre-publication history

The pre-publication history for this paper can be accessed here:

http://www.biomedcentral.com/1471-244X/13/290/prepub

## References

[B1] Hudson J Hiripi E Pope H Kessler R The prevalence and correlates of eating disorders in the national comorbidity survey replication Biol Psych 2007 61 348 358 10.1016/j.biopsych.2006.03.040 PMC1892232 16815322

[B2] Fitzpatrick K Lock J Anorexia nervosa Clin Evid (Online) 2011 April 11 PMC3275304 21481284

[B3] Swanson S Crow S Le Grange D Swendsen J Merikangas K Prevelance and correlates of eating disorders in adolecents: results from the national comorbidity survey replication adolescent supplement Arch Gen Psychiatry 2011 68 7 714 723 10.1001/archgenpsychiatry.2011.22 21383252 PMC5546800

[B4] American Psychiatric Association Treatment of patients with eating disorders, 3rd ed Am J Psychiatry 2006 163 Suppl 4 54 16925191

[B5] Mehler PS Krantz M Anorexia nervosa medical issues J Womens Health 2003 12 4 331 340 10.1089/154099903765448844 12804340

[B6] Bruch H Anorexia nervosa: therapy and theory Am J Psychol 1982 139 1531 1538 10.1176/ajp.139.12.1531 6816075

[B7] Fassino S Piero A Tomba E Abbate-Daga G Factors associated with dropout from treatment for eating disorders: a comprehensive literature review BMC Psychiatry 2009 9 67 10.1186/1471-244X-9-67 19818137 PMC2765944

[B8] Steinhausen HC The outcome of anorexia nervosa in the 20th century Am J Psychiatry 2002 159 8 1284 1293 10.1176/appi.ajp.159.8.1284 12153817

[B9] Pike K Long-term course of anorexia nervosa: response, relapse, remission, and recovery Clin Psychol Rev 1998 18 447 475 10.1016/S0272-7358(98)00014-2 9638357

[B10] Steinglass J Sysko R Mayer L Berner L Schebendach J Wang Y Chen H Albano A Simpson H Walsh B Pre-meal anxiety and food intake in anorexia nervosa Appetite 2010 55 2 214 218 10.1016/j.appet.2010.05.090 20570701 PMC2939314

[B11] Kaye W Fudge J Paulus M New insight into symptoms and neurocircuit function of anorexia nervosa Nat Rev Neurosci 2009 10 8 573 584 10.1038/nrn2682 19603056 PMC13038070

[B12] Bardone-Cone A Fitzsimmons-Craft E Harney M Maldonado C Lawson M Smith R Robinson D The inter-relationships between vegetarianism and eating disorders among females J Acad Nutr Diet 2012 112 8 1247 1252 10.1016/j.jand.2012.05.007 22818732 PMC3402905

[B13] Gwirtsman H Kaye W Curtis S Lyter L Energy intake and dietary macronutrient content in women with anorexia nervosa and volunteers J Am Diet Assoc 1989 89 1 54 57 2909592

[B14] Holman R Adams C Nelson R Grater S Jaskiewicz J Johnson S Erdman JJ Patients with anorexia nervosa demonstrate deficiencies of selected essential fatty acids, compensatory changes in nonessential fatty acids and decreased fluidity of plasma lipids J Nutr 1995 125 4 901 907 7722693 10.1093/jn/125.4.901

[B15] Schebendach J Mayer L Devlin M Attia E Cotento I Wolf R Walsh B Dietary energy density and diet variety as predictors of outcome in anorexia nervosa Am J Clin Nutr 2008 87 4 810 816 18400701 10.1093/ajcn/87.4.810

[B16] Kaye W Gwirtsman H Obarzanek E George T Jimerson DC Ebert MH Caloric intake necessary for weight maintenance in anorexia nervosa: nonbulimics require greater caloric intake than bulimics Am J Clin Nutr 1986 44 435 443 3766430 10.1093/ajcn/44.4.435

[B17] Walker J Roberts S Halmi K Goldberg S Caloric requirements for weight gain in anorexia nervosa Am J Clin Nutr 1979 32 7 1396 1400 453054 10.1093/ajcn/32.7.1396

[B18] Weltzin TE Fernstrom MH Hansen D McConaha C Kaye WH Abnormal caloric requirements for weight maintenance in patients with anorexia and bulimia nervosa Am J Psychiatry 1991 148 12 1675 1682 1957930 10.1176/ajp.148.12.1675

[B19] Nova E Varela P Lopez-Vidriero I Toro O Cenal M Casas J Marcos A A one-year follow-up study in anorexia nervosa. dietary pattern and anthropometrical evolution Eur J Clin Nutr 2001 55 7 547 554 10.1038/sj.ejcn.1601181 11464228

[B20] Misra M Tsai P Anderson E Hubbad J Gallagher K Soyka L Miller K Herzog D Klibanski A Nutrient intake in community-dwelling adolescent girls with anorexia nervosa and in healthy adolescents Am J Clin Nutr 2006 84 4 698 706 17023694 10.1093/ajcn/84.4.698 PMC3210565

[B21] Affenito S Dohm F Crawford P Daniels S Striegel-Moore R The national heart, lung, and blood institute growth and health study J Pediatr 2002 141 5 701 705 10.1067/mpd.2002.129840 12410201

[B22] Fernstrom M Weltzin T Neuberger S Srinivasagam N Kaye W Twenty-four hour food intake in patients with anorexia nervosa and in healthy control subjects Biol Psych 1994 36 696 702 10.1016/0006-3223(94)91179-7 7880939

[B23] Hadigan C Anderson E Miller K Hubbard J Herzog D Klibanski A Grinspoon S Assessment of macronutrient and micronutrient intake in women with anorexia nervosa Int J Eat Disord 2000 28 284 292 10.1002/1098-108X(200011)28:3<284::AID-EAT5>3.0.CO;2-G 10942914

[B24] Drewnowski A Pierce B Halmi K Fat aversion in eating disorders Appetite 1988 10 119 131 10.1016/0195-6663(88)90063-3 3164990

[B25] Sunday S Halmi K Energy intake and body composition in anroexia and bulimia nervosa Physiol Behav 2003 78 1 11 17 10.1016/S0031-9384(02)00879-X 12536005

[B26] Huse DM Lucas AR Dietary patterns in anorexia nervosa Am J Clin Nutr 1984 40 2 251 254 6465058 10.1093/ajcn/40.2.251

[B27] Micali N Northstone K Emmett P Naumann U Treasure J Nutritional intake and dietary patterns in pregnancy: a longitudinal study of women with lifetime eating disorders Br J Nutr 2012 108 11 2093 2099 10.1017/S0007114512000256 22784642

[B28] Van Binsbergen C Hulshof K Wedel M Odink J Coelingh Bennink H Food preferences and aversions and dietary pattern in anorexia nervosa patients Eur J Clin Nutr 1988 42 8 671 678 3181100

[B29] Yackobovich-Gavan M Golan M Valevski A Kreitler S Bachar E Lieblich A Mitrani E Weizman A Stein D An integrative quantitative model of factors influencing the course of anorexia nervosa over time Int J Eat Disord 2009 42 306 317 10.1002/eat.20624 19040269

[B30] Fontana L Klein S Holloszy J Premachandra B Effect of long-term calorie restriction with adequate protein and micronutrients on thyroid hormones J Clin Endocrinol Metab 2006 91 8 3232 3235 10.1210/jc.2006-0328 16720655

[B31] Beaumont P Chambers T Rouse L Abraham S The diet composition and nutritional knowledge of patients with anorexia nervosa J Human Nutr 1981 35 265 273 7276554 10.3109/09637488109143052

[B32] Russell G The nutritional disorder in anorexia nervosa J Psychosom Res 1967 11 141 149 10.1016/0022-3999(67)90066-9 6049025

[B33] Jauregui Lobera I Bolanos Rios P Choice of diet in patients with anorexia nervosa Nutr Hosp 2009 24 6 682 687 20049371

[B34] Schenbendach J Mayer L Devlin M Attia E Walsh B Dietary energy density and diet variety as risk factors for relapse in anorexia nervosa: a replication Int J Eat Disord 2012 45 1 79 84 10.1002/eat.20922 21448937 PMC4469286

[B35] Petersen R Kaye W Gwirtsman H Comparison of calculated estimates and laboratory analysis of food offered to hospitalized eating disorder patients J Am Diet Assoc 1986 86 4 490 492 3958399

[B36] Mehanna H Nankivell P Moledina J Travis J Refeeding syndrome–awareness, prevention and management Head Neck Oncol 2009 26 1 4 10.1186/1758-3284-1-4 PMC2654033 19284691

[B37] Adkins S Recognizing and preventing refeeding syndrome Dimens Crit Care Nurs 2009 28 2 53 58 quiz 59–60 19225312 10.1097/DCC.0b013e318195d3e0

[B38] Yantis M Velander r: how to recognize and respond to refeeding syndrome Nursing 2008 38 5 34 39 quiz 39–40 10.1097/01.NURSE.0000317679.01914.a4 18431199

[B39] NICE Core interventions in the treatment and management of anorexia nervosa, bulimia nervosa and related eating disorders (Clinical Guideline 9) 2004 London: National Collaborating Centre for Medical Health

[B40] Guarda A Treatment of anorexia nervosa: insights and obstacles Physiol Behav 2008 94 1 113 120 10.1016/j.physbeh.2007.11.020 18155737

[B41] Halmi K Pragmatic informtion on the eating disorders Psychiatr Clin North Am 1982 5 2 371 377 6750574

[B42] Rock C Curran-Celentano J Nutritional managment of eating disorders Psychitr Clin N orth Am 1996 19 4 701 713 10.1016/S0193-953X(05)70376-2 8933603

[B43] Gaudiani J Sabel A Mascolo MM PS Severe anorexia nervosa: outcomes from a medical stabilization unit Int J Eat Disord 2012 45 1 85 92 10.1002/eat.20889 22170021

[B44] Gentile M Enteral nutrition for feeding severely underfed patients with anorexia nervosa Nutrients 2012 4 9 1293 1303 23112917 10.3390/nu4091293 PMC3475239

[B45] Kaye W Gwirtsman H Obarzanek E George D Relative importance of calorie intake needed to gain weight and level of physical activity in anorexia nervosa Am J Clin Nutr 1988 47 989 994 3376913 10.1093/ajcn/47.6.989

[B46] Setnick J Micronutrient deficiencies and supplementation in anorexia and bulimia nervoa: a review of literature Nutr Clin Pract 2010 25 2 137 142 10.1177/0884533610361478 20413694

[B47] Kohn MR Golden N Eating disorders in children and adolescents: epidemiology, diagnosis and treatment Paediatr Drugs 2001 3 2 91 99 10.2165/00128072-200103020-00002 11269642

[B48] Seidenfeld M Sosin E Rickert V Nutrition and eating disorders in adolescents Mt Sinai J Med 2004 71 155 161 15164127

[B49] Whitelaw M Gilbertson H Lam P Sawyer S Does aggressive refeeding in hospitalized adolescents with anorexia nervosa result in increased hypophosphatemia? J Adolesc Health 2010 46 6 577 582 10.1016/j.jadohealth.2009.11.207 20472215

[B50] Katzman D Refeeding hospitalized adolescents with anorexia nervosa: is "start low, advance slow" urban legend or evidence based? J Adolesc Health 2012 50 1 1 2 10.1016/j.jadohealth.2011.10.003 22188827

[B51] Norrington A Stanley R Tremlett M Birrell G Medical management of acute severe anorexia nervosa Arch Dis Child Educ Pract Ed 2012 92 2 48 54 21764823 10.1136/adc.2010.199885

[B52] Mehler P Weiner K Anorexia nervosa and total parenteral nutrition Int J Eat Disord 1993 14 3 297 304 10.1002/1098-108X(199311)14:3<297::AID-EAT2260140308>3.0.CO;2-X 8275066

[B53] Golden N Meyer W Nutritional rehabilitation of anorexia nervosa. goals and dangers Int J Adolesc Med Health 2004 16 2 131 144 15266992 10.1515/ijamh.2004.16.2.131

[B54] Kohn M Madden S Clarke S Refeeding in anorexia nervosa: increased safety and efficiency through understanding the pathophysiology of protein calorie malnutrition Curr Opin Pediatr 2011 23 4 390 394 10.1097/MOP.0b013e3283487591 21670680

[B55] Dempsey DT Crosby LO Pertschuk MJ Feurer ID Buzby GP Mullen JL Weight gain and nutritional efficacy in anorexia nervosa Am J Clin Nutr 1984 39 2 236 242 6421142 10.1093/ajcn/39.2.236

[B56] Newman M Halmi K Marchi P Relationship of clinical factors to caloric requirements in subtypes of eating disorders Biol Psychiatry 1987 22 1253 1263 10.1016/0006-3223(87)90033-3 3478097

[B57] Forbes G Kreipe R Lipinski B Body composition and the energy cost of weight gain Hum Nutr Clin Nutr 1982 36 6 485 487 7161143

[B58] Russell G Mezey A An analysis of weight gain in patients with anorexia nervosa treated with high calorie diets Clin Sci 1962 23 449 461 13975640

[B59] Mehler P Winkelman A Andersen DG JL Nutritional rehabilitation: practical guidelines for refeeding the anorectic patient J Nutr Metab 2010 10.1155/2010/625782 PMC2925090 20798756

[B60] Rosenbaum M Hirsch J Gallagher D Eibel R Long-term persistence of adaptive thermogenesis in subjects who have maintained a reduced body weight Am J Clin Nutr 2008 88 4 906 912 18842775 10.1093/ajcn/88.4.906

[B61] Rosenbaum MK HR Mayer L Hirsch J Leibel R Energy intake in weight reduced humans Brain Res 2010 1350 95 102 20595050 10.1016/j.brainres.2010.05.062 PMC2926239

[B62] Reiter C Graves L Nutrition therapy for eating disorders Nutr Clin Pract 2010 25 2 122 136 10.1177/0884533610361606 20413693

[B63] Moukaddem M Boulier A Apfelbaum M Rigaud D Increase in diet-induced thermogenesis at the start of refeeding in severely malnourished anorexia nervosa patients Am J Clin Nutr 1997 66 1 133 140 9209181 10.1093/ajcn/66.1.133

[B64] Kron L Katz JL Gorzynski G Weiner H Hyperactivity in anorexia nervosa: a fundamental clinical feature Compr Psychiatry 1978 19 5 433 440 10.1016/0010-440X(78)90072-X 679677

[B65] Vaisman N Rossi M Corey M Clarke R Goldberg E Pencharz P Effect of refeeding on the energy metabolism of adolescent girls who have anorexia nervosa Eur J Clin Nutr 1991 45 11 527 537 1782924

[B66] Kurpad A Kulkarni R Shetty P Reduced thermoregulatory thermogenesis in undernutrition Eur J Clin Nutr 1989 43 1 27 33 2731495

[B67] Rigaud D Verges B Colas-Linhart N Petiet A Moukkaddem M Van Wymelbeke V Brondel L Hormonal and psychological factors linked to the increased thermic effect of food in malnourished fasting anorexia nervosa J Clin Endocrinol Metab 2007 92 5 1623 1629 10.1210/jc.2006-1319 17341571

[B68] Stordy BM V Kalucy RC AH Weight gain, thermic effect of glucose and resting metabolic rate during recovery from anorexia nervosa Am J Clin Nutr 1977 30 2 138 146 835500 10.1093/ajcn/30.2.138

[B69] Kaye WH Gwirtsman HE Lake CR Siever LJ Jimerson DC Ebert MH Murphy DL Disturbances of norepinephrine metabolism and alpha-2 adrenergic receptor activity in anorexia nervosa: relationship to nutritional state Psychopharmacol Bull 1985 21 3 419 423 2994154

[B70] Landsberg LY JB The role of the sympathetic nervous system and catecholamines in the regulation of energy metabolism Am J Clin Nutr 1983 38 6 1018 1024 6359855 10.1093/ajcn/38.6.1018

[B71] O'Dea K Esler M Leonard P Stockigt J Nestel P Noradrenaline turnover during under- and overeating in normal weight subjects Metabolism 1982 31 869 896 10.1016/0026-0495(82)90178-0 7121260

[B72] Casper R Carbohydrate metabolism and its regulatory hormones in anorexia nervosa Psy Res 1996 62 1 85 96 10.1016/0165-1781(96)02984-8 8739118

[B73] Yamashita S Kawai KY T Inoo T Yokoyama H Morita C Takii M Kubo C BMI, body composition, and the energy requirement for body weight gain in patients with anorexia nervosa Int J Eat Disord 2010 43 4 365 371 19459214 10.1002/eat.20700

[B74] Huas C Godart N Foulon C Pham-Scottez A Divac S Fedorowicz V Peyracque E Dardennes R Falissard B Rouillon F Predictors of dropout from inpatient treatment for anorexia nervosa: data from a large French sample Psychiatry Res 2011 185 3 421 426 10.1016/j.psychres.2009.12.004 20546922

[B75] Mayer L Roberto C Glasofer D Etu S Gallagher D Wang J Heymsfield S Pierson RJ Attia E Devlin M Does percent body fat predict outcome in anorexia nervosa? Am J Psychiatry 2007 164 6 970 972 10.1176/appi.ajp.164.6.970 17541059 PMC2741391

[B76] Hebebrand J Himmelmann G Herzog W Herpertz-Dahlmann B Steinhausen H Amstein MS R Deter H Remschmidt H Schafer H Prediction of low body weight at long-term follow-up in acute anorexia nervosa by low body weight at referral Am J Psychiatry 1997 154 4 566 569 9090350 10.1176/ajp.154.4.566

[B77] Mitchell J Crow S Medical complications of anorexia nervosa and bulimia nervosa Curr Opin Psychiatry 2006 19 4 438 443 10.1097/01.yco.0000228768.79097.3e 16721178

[B78] Vignaud M Constantin J Ruivard M Villemeyre-Plane M Futier E Bazin J Annane D AZUREA Group (AnorexieRea Study Group) Refeeding syndrome influences outcome of anorexia nervosa patients in intensive care unit: an observational study Crit Care 2010 14 5 R172 10.1186/cc9274 20920160 PMC3219274

[B79] Langan S Farrell P Vitamin E, vitamin A and essential fatty acid status of patients hospitalized for anorexia nervosa Am J Clin Nutr 1985 41 5 1054 1060 3993608 10.1093/ajcn/41.5.1054

[B80] Attia E Walsh B Behavioral management for anorexia nervosa New Eng J Med 2009 360 5 500 506 10.1056/NEJMct0805569 19179317

[B81] Hearing S Refeeding syndrome BMJ 2004 328 7445 908 909 10.1136/bmj.328.7445.908 15087326 PMC390152

[B82] Helweg-Larsen P Hoffmeyer H Kieleir J Thaysen E Thaysen J Thygesen P Wulff M Famine disease in German concentration camps: complications and sequels 144 1952 274 Suppl 81 92 178–198

[B83] McLoughlin D Spargo E Wassif W Newham D Peters T Lantos P Russell G Structural and functional changes in skeletal muscle in anorexia nervosa Acta Neuropathol 1998 95 6 632 640 10.1007/s004010050850 9650756

[B84] Winter T The effects of undernutrition and refeeding on metabolism and digestive function Curr Opin Clin Nutr Metab Care 2006 9 5 596 602 10.1097/01.mco.0000241670.24923.5b 16912556

[B85] Diamanti A Basso M Cecchetti C Monti L Noto C De Maria F Castro M Digestive complication in severe malnourished anorexia nervosa patient: a case report of necrotizing colitis Int J Eat Disord 2011 44 1 91 93 10.1002/eat.20778 19950113

[B86] Mitrany E Melamed Y Compulsory treatment of anorexia nervosa Is J Psychiatry Relat Sci 2005 42 3 185 190 16335631

[B87] Tan J Stewart A Fitzpatrick R Hope T Attitudes of patients with anorexia nervosa to compulsory treatment and coercion Int J Law Psychiatry 2010 33 1 13 19 10.1016/j.ijlp.2009.10.003 19926134 PMC2808473

[B88] Vitousek K Watson S Wilson G Enhancing motivation for change in treatment-resistant eating disorders Clin Psychol Rev 1998 18 4 391 420 10.1016/S0272-7358(98)00012-9 9638355

[B89] Channon S DeSilva W Psychological correlates of weight gain in patients with anoreixa nervosa J Psychiatr Res 1985 19 267 271 10.1016/0022-3956(85)90027-5 4045744

[B90] Otten J Hellwig J Meyers L DRI: dietary reference intakes: the essential guide to nutrient requirements 2006 Washington DC: National Academies Press

[B91] Lutter C Rivera J Nutritional status of infants and young children and characteristics of their diets J Nutr 2003 133 9 2941 2949 10.1093/jn/133.9.2941S 12949391

[B92] Duerksen D McCurdy K Essential fatty acid deficiency in a severely malnourished patient receiving parenteral nutrition Dig Dis Sci 2005 50 12 2386 2368 10.1007/s10620-005-3068-9 16416195

[B93] Jáuregui-Garrido B Bolaños-Ríos P Santiago-Fernandez M Jauregui-Lobera I Lipid profile and cardiovascular risk in anorexia nervosa; the effect of nutritional treatment Nutr Hosp 2012 27 3 908 913 23114953 10.3305/nh.2012.27.3.5752

[B94] Rosell ML-W Z Appleby P Sanders T Allen N Key T Long-chain n-3 polyunsaturated fatty acids in plasma in British meat-eating, vegetarian, and vegan men Am J Clin Nutr 2005 82 2 327 334 16087975 10.1093/ajcn.82.2.327

[B95] Ayton AA A Horrobin D A pilot open case series of ethyl-EPA supplementation in the treatment of anorexia nervosa Prostaglandins Leukot Essent Fatty Acids 2004 71 4 205 209 10.1016/j.plefa.2004.03.007 15301789

[B96] Abell T Malagelada J Lucas A Brown M Camilleri M Go V Azpiroz F Callaway C Kao P Zinsmeister A Gastric electromechanical and neurohormonal function in anorexia nervosa Gastroenterology 1987 93 5 958 965 3653645 10.1016/0016-5085(87)90557-9

[B97] McCallum R Grill B Lange R Plankly M Glass E Greenfeld D Definition of a gastric emptying abnormality in patients with anorexia nervosa Dig Dis Sci 1985 30 8 713 722 10.1007/BF01320484 4017831

